# Supplementary material for: Active self-assembly of piezoelectric biomolecular films via synergistic nanoconfinement and in-situ poling
Source: Nat Commun. 2023 Jul 11;14:4094. doi: 10.1038/s41467-023-39692-y (PMC10336032; doi:10.1038/s41467-023-39692-y)
Supplement: Supplementary file 1 — Supplementary Information [file 41467_2023_39692_MOESM1_ESM.pdf]

Supplementary Information for

Active self-assembly of piezoelectric biomolecular films via synergistic nanoconfinement and *in-situ* poling

AUTHORS

Zhuomin Zhang[1,2†], Xuemu Li[1,2†], Zehua Peng[1,2], Xiaodong Yan[1,2], Shiyuan Liu[1,2], Ying Hong[1,2], Yao Shan[1,2], Xiaote Xu[1,2], Lihan Jin[2], Bingren Liu[2], Xinyu Zhang[3], Yu Chai[3], Shujun Zhang[4]*, Alex K.-Y. Jen[5,6,7,8]*, Zhengbao Yang[1,2,5]*

AFFILIATIONS

1. Department of Mechanical and Aerospace Engineering, Hong Kong University of Science and Technology, Clear Water Bay, Hong Kong, China
2. Department of Mechanical Engineering, City University of Hong Kong, Hong Kong, China
3. Department of Physics, City University of Hong Kong, Hong Kong, China
4. Institute for Superconducting and Electronic Materials, Australian Institute of Innovative Materials, University of Wollongong, Wollongong, New South Wales, Australia
5. Department of Materials Science and Engineering, City University of Hong Kong, Kowloon, Hong Kong
6. Department of Chemistry, City University of Hong Kong, Kowloon, Hong Kong
7. Hong Kong Institute for Clean Energy, City University of Hong Kong, Kowloon, Hong Kong
8. Department of Materials Science and Engineering, University of Washington, Seattle, WA 98195-2120 USA

† These authors contributed equally to this work

* Correspondence to: shujun@uow.edu.au; alexjen@cityu.edu.hk; zbyang@ust.hk

This PDF file includes:

Supplementary Notes 1 to 2
Supplementary Figures 1 to 26
Supplementary Tables 1 to 4
Supplementary References

Supplementary Notes

S1. The principle and theoretical analysis of electrohydrodynamic jetting

The dynamic behavior of the electrofluid is affected by the combination of various forces such as surface tension, hydrodynamic force, viscous force, and gravity (Figure S1). The mechanism of the jet formation process is essential for optimizing electrohydrodynamic spray parameters and achieving stable jetting and high-quality films. The formation process of an electrohydrodynamic jet is calculated using a set of equations in which electrical and mechanical forces are included[1],

$$\frac{\partial \rho_i}{\partial t} + \nabla \cdot (\rho_i v_i) = \sum_{j=1}^{n} I_{ij}, \qquad (1)$$

$$\frac{\partial \rho_i v_i}{\partial t} + \nabla \cdot (\rho_i v_i \otimes v_i) = \nabla \cdot \prod_{\ell} + \rho_i g + \sum_{j=1}^{n} P_{ij} + L_{\ell} \qquad (2)$$

where i refers to the gas or liquid phase. The symbol $\otimes$ is the dyadic product of the vectors. The stress tensor on the liquid surface is as follows,

$$\prod_{\ell} = Grad(lp) + \eta_{\ell} \nabla v_{\ell} \qquad (3)$$

where p is the dynamic pressure and η is the liquid viscosity. *Grad* is the different operator on a scalar. This difference is due to the pressures on both sides of the interfacial surface ($Grad(p) = p_{\ell} - p_g$). Then the force density on the liquid jet can be obtained depending on Eq. (3),

$$\frac{\partial \rho_{\ell}}{\partial \mathrm{t}} = \rho_{\ell} g + L_{\ell} - \phi_{st} - \nabla \cdot (Grad(lp)) + \eta_{\ell} \nabla v_{\ell} + \rho_{\ell} v_{\ell} \otimes v_{\ell} \qquad (4)$$

During the spray process, the droplets may be broken down into tiny satellite droplets, which is desirable to obtain the nanoscale particles. For our glycine solution, the electric field force, the repulsive Coulomb force, and the downward or upward contraction forces caused by surface tension are the main forces that determine the size and moving direction of the droplets. The diameter of the initial droplet is[2]

$$D_d = G(k)(\frac{Q \kappa \varepsilon_0}{K})^{1/3} \qquad (5)$$

where $G(k) = 10.87\kappa^{-6/5} + 4.08\kappa^{-1/3}$, Q is the flow rate of ink, k is the dielectric constant, ε_0 is the permittivity of vacuum, and K is the liquid electrical conductivity. So, the droplet size can be influenced by the flow rate and properties of the solution. In the process of droplets falling, their size is gradually decreased due to solvent evaporation. It is essential to form continuous wet precursor films with nanodroplets to achieve smooth and dense films. For this purpose, the deposited droplets should remain fluidic to allow the droplets to merge into a continuous wet film.

S2. Melting point depression effects of β-glycine nanosized crystals

In the critical size region, it is expected that the thermotropic properties of crystals should be strongly related to sizes because the absolute values of the volume free energy and the surface free energy are comparable. It has been demonstrated in ice[6], metals[7], and organic solids[8] that the melting points and enthalpies of fusion are shifted greatly due to the crystals confinement. Their specific crystal properties were uncovered by the crystallization in nanoporous media. The thermotropic properties of crystals are affected by the nanoconfinement, and can be expressed by the classical Gibbs−Thomson equation:

$$\frac{\Delta T_{melt}}{T_{bulk}} = -\frac{2M}{\Delta H \rho r}\gamma \cos\theta \qquad (6)$$

in which M is the molecular mass of the compound comprising the particle, ρ denotes the particle density, r represents the particle radius (assuming spherical shape), γ is the specific interfacial energy (surface tension) between the solid phase and the fluid surrounding it, θ is the interfacial angle between the condensed phase and any phase it may have nucleated upon, ΔH is the molar heat of fusion of the bulk condensed phase, $T_{melt}(r)$ is the melting temperature of the condensed phase of radius r, and T_{bulk} is the melting temperature of the condensed phase in the bulk state.

The Gibbs−Thomson equation predicts a linear inverse relationship between ΔT_{melt} and the crystal size r, assuming other parameters are constant and independent of the particle size. The contact angle θ is often assumed to be 180° for the homogeneous nucleation. The influence of size on polymorphism could be expressed by a simplified version of the Gibbs−Thomson equation as follows:

$$\frac{\Delta T_{melt}}{T_{bulk}} = -\frac{2M}{\Delta H \rho r}\gamma \qquad (7)$$

Based on this relationship, the melting point should be decreased as the crystals' size is reduced. It can be inferred that the β-glycine nanocrystalline films should have thermotropic properties different from their corresponding bulk forms, which is consistent with our experiment results.

Supplementary Figures

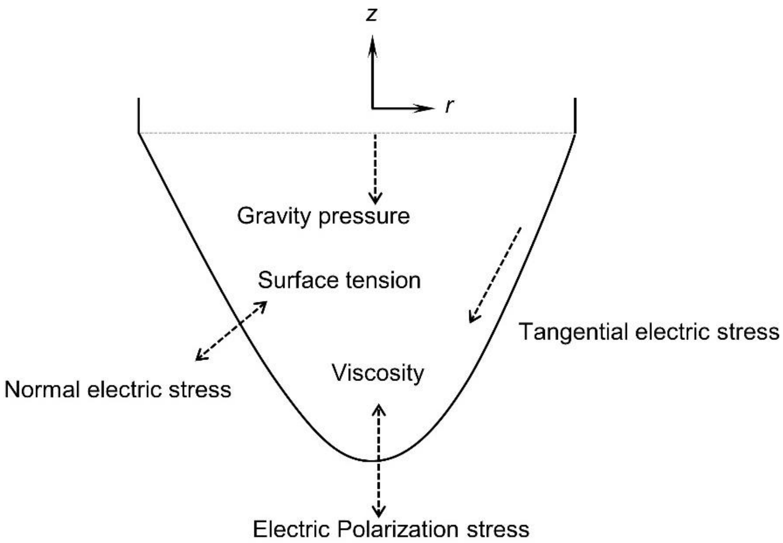

Supplementary Figure 1. Schematic showing the forces acting on the fluid surface during the process of electrohydrodynamically induced jet formation.

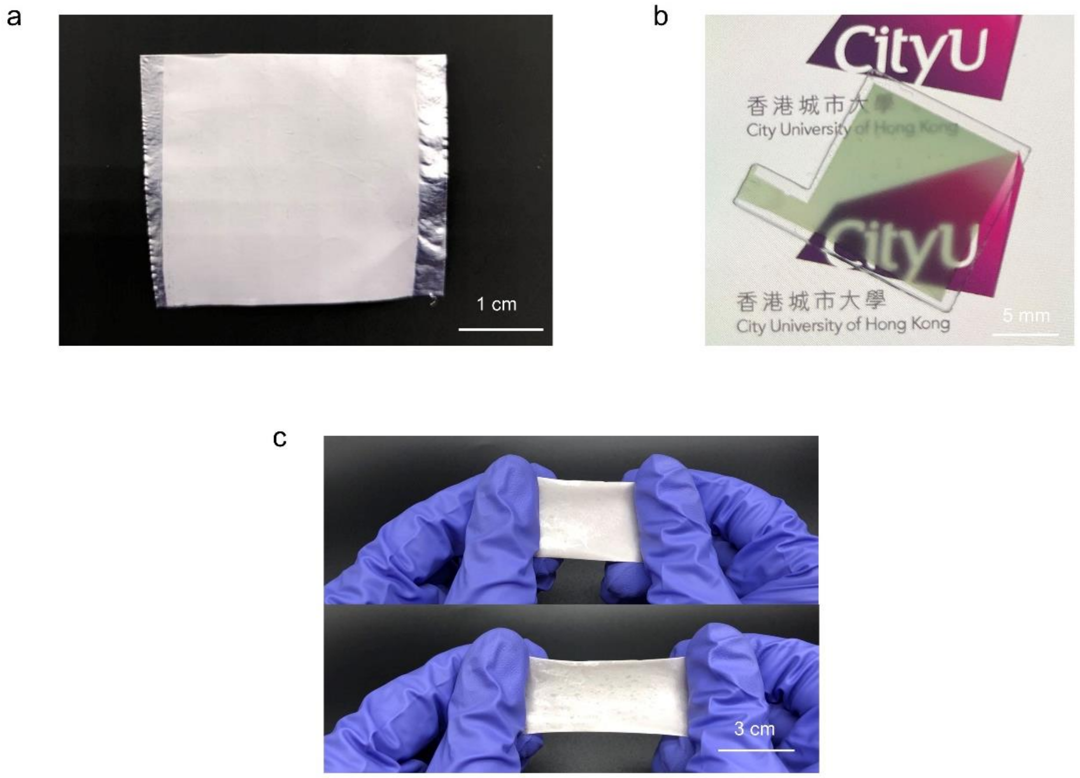

Supplementary Figure 2. Photographs of a β-glycine nanocrystalline film on an aluminium foil (a), film on a gold-coated PMMA substrate (b), and film on a platinum-coated PDMS substrate (c), initial state (top) and stretched state (bottom).

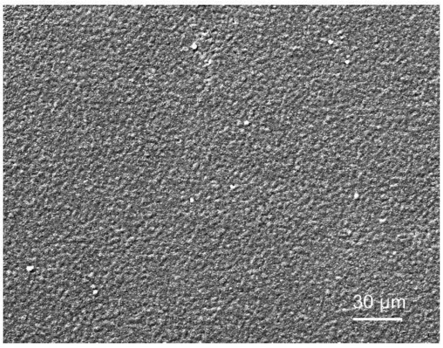

Supplementary Figure 3. Surface topography SEM image of the β-glycine nanocrystalline film at a larger scale showing the uniform distribution of compact nanosized grains.

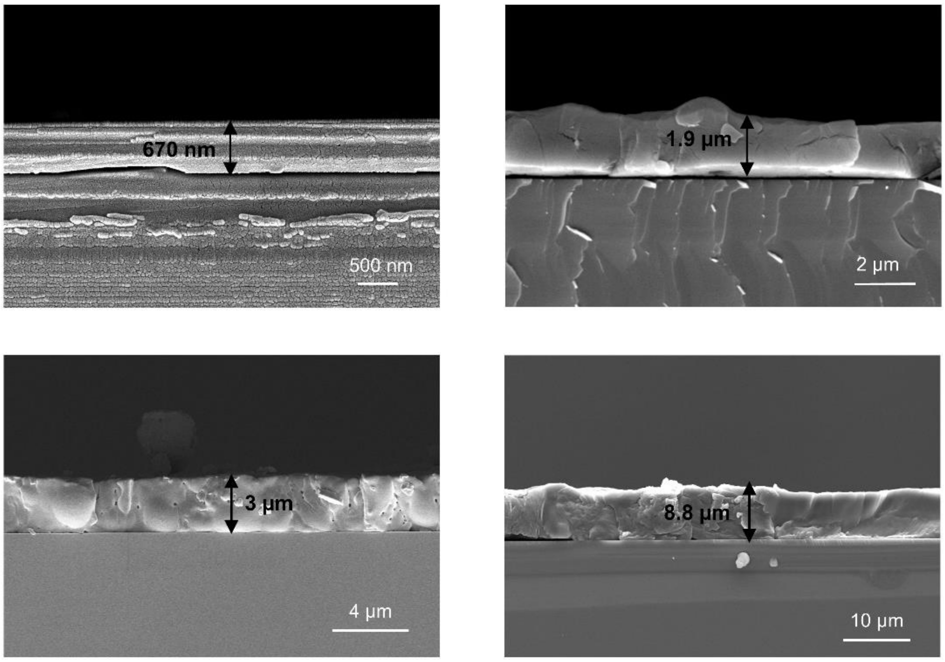

Supplementary Figure 4. Cross-sectional SEM image of β-glycine nanocrystalline films with variable thicknesses of 670 nm, 1.9 μm, 3 μm, and 8.8 μm, respectively.

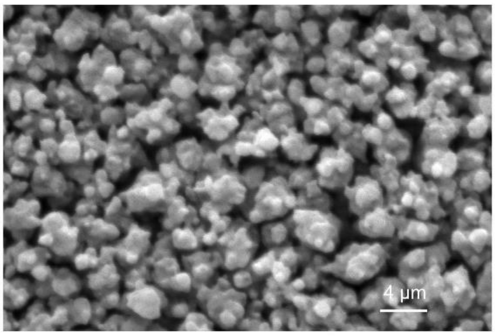

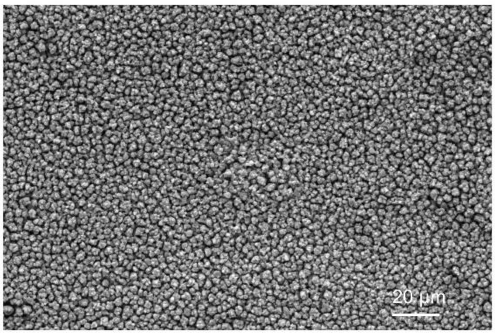

Supplementary Figure 5. Surface topography SEM image of the films obtained by introducing the *in-situ* heating into the synthesis process showing the hollow and loose structure, evidencing the effect of water shell of nanograins on compact film formation.

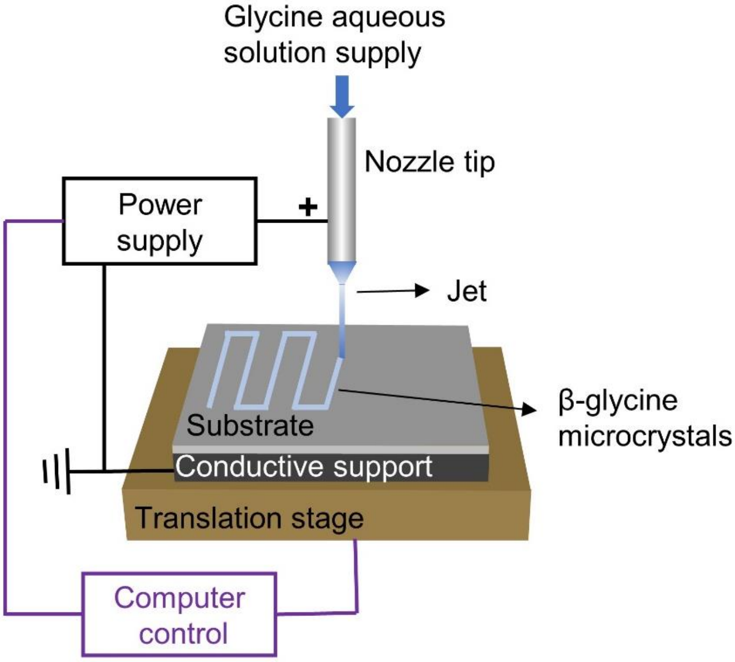

Supplementary Figure 6. Schematic of the electrohydrodynamic focusing deposition process for creating the β-glycine microcrystals.

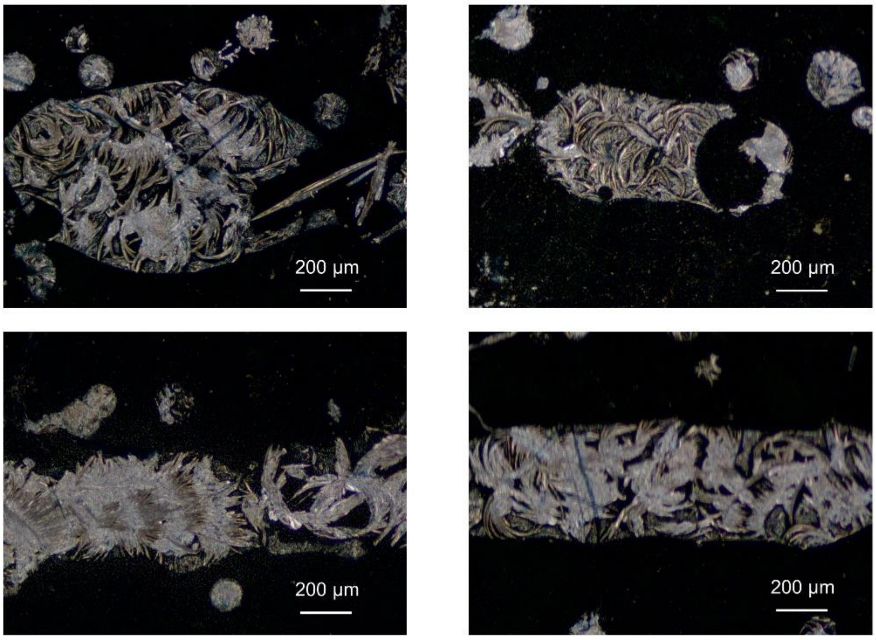

Supplementary Figure 7. Optical images of β-glycine microcrystals obtained by the electrohydrodynamic focusing deposition instead of electrohydrodynamic spray.

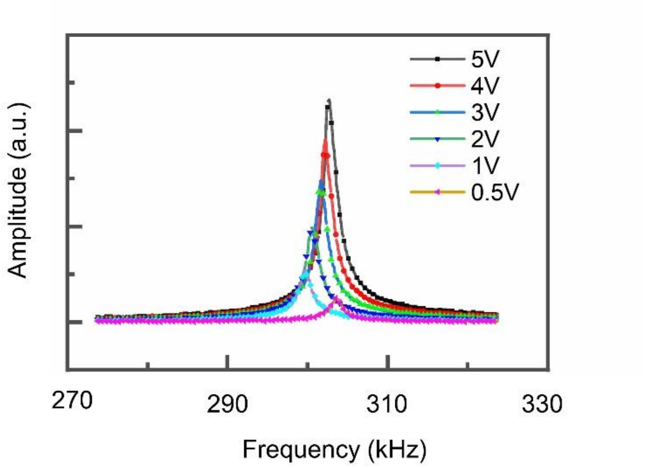

Supplementary Figure 8. PFM OOP responses of β-glycine nanocrystalline films across resonant frequency with different applied voltages.

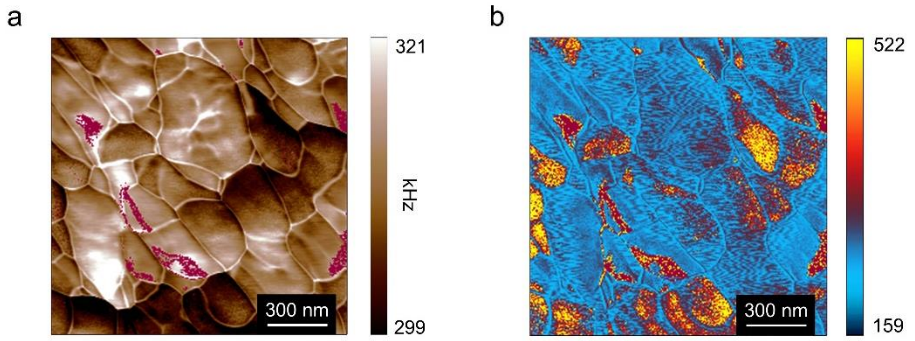

Supplementary Figure 9. Resonant frequency distribution mapping (a) and quality factor distribution mapping (b) of β-glycine nanocrystalline films in a 1.5 × 1.5 um^2 area.

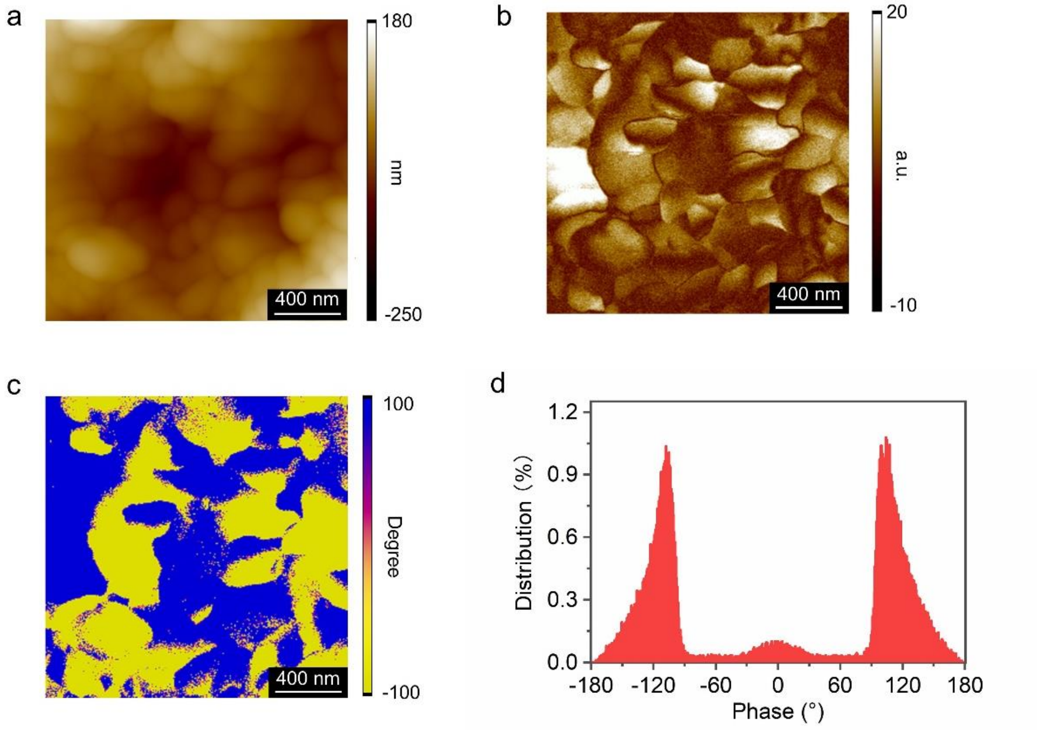

Supplementary Figure 10. Lateral PFM measurements of β-glycine nanocrystalline films. a, AFM surface topography mapping. b, PFM IP amplitude mapping. c, IP phase mapping. d, Histogram calculated from the phase mapping in c. The applied AC voltage is 10 V.

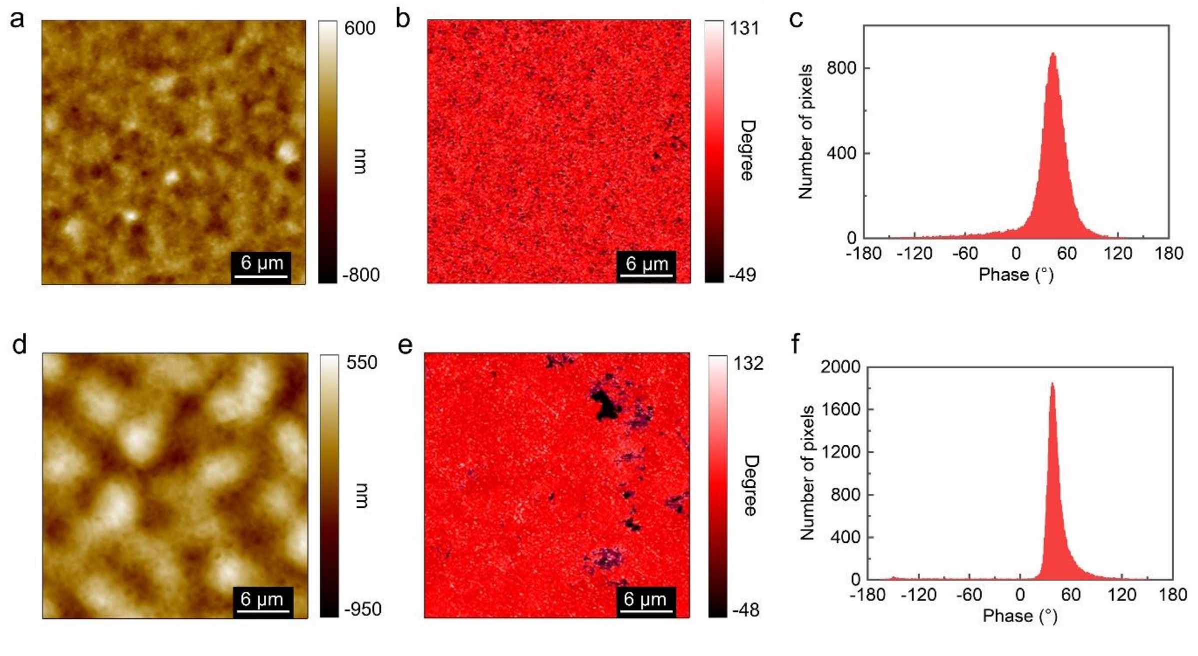

Supplementary Figure 11. Large-area PFM measurements of β-glycine nanocrystalline films. a, AFM surface topography mapping. b, PFM OOP phase mapping. c, Histogram calculated from the phase mapping in b. d, AFM surface topography mapping in another area. e, The corresponding PFM OOP phase mapping. f, Histogram calculated from the phase mapping in e.

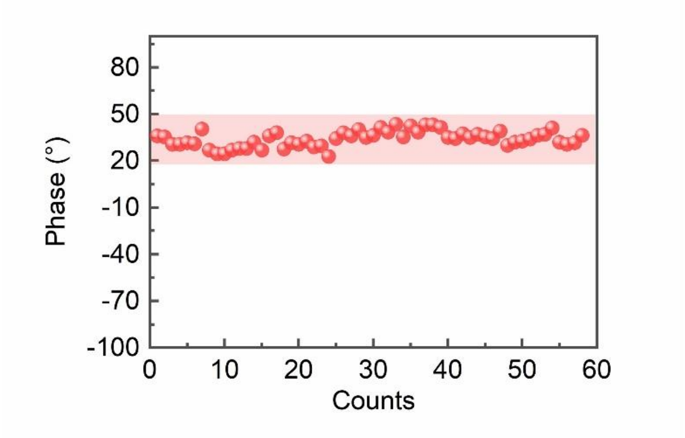

Supplementary Figure 12. The PFM OOP phase data of randomly selected regions from different samples showing a uniform and consistent value, indicating that the polarization of the entire film is in the direction of the applied electric field.

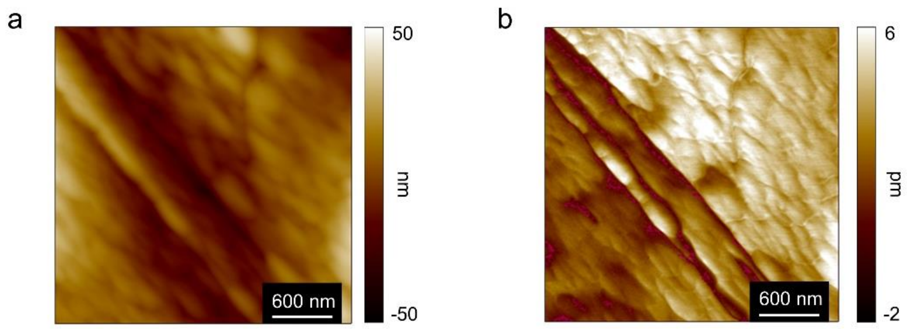

Supplementary Figure 13. PFM measurements of β-glycine microcrystals obtained by electrohydrodynamic focus deposition. a, AFM surface topography mapping. b, PFM OOP amplitude mapping.

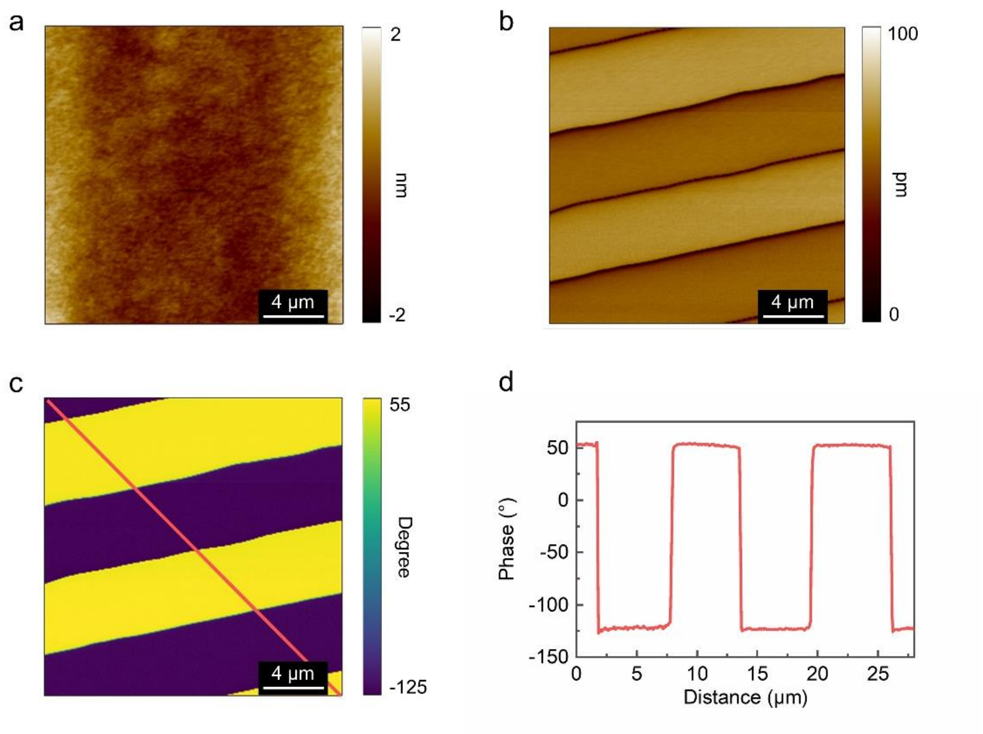

Supplementary Figure 14. PFM measurements of standard PPLN sample. a, AFM surface topography mapping. b, PFM OOP amplitude mapping. c, OOP phase mapping. d, Cross-sectional profile of piezoelectric polarization responses along the red line in phase mapping in c.

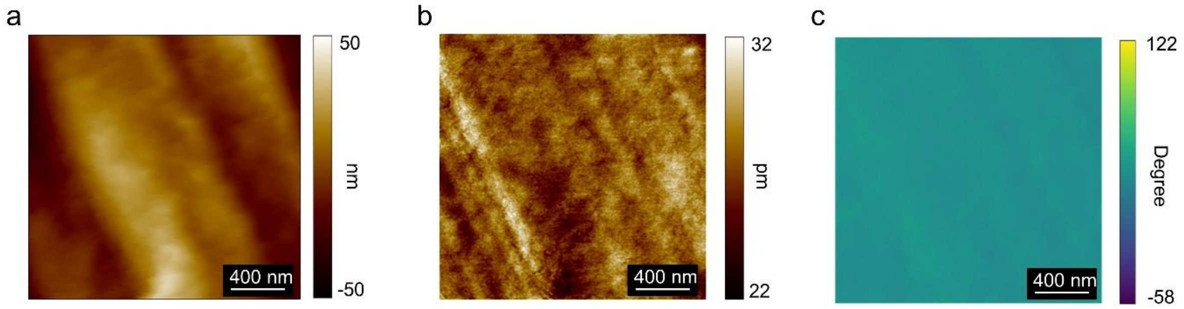

Supplementary Figure 15. PFM measurements of commercial PVDF thin film (thickness: 28 μm). a, AFM surface topography mapping. b, PFM OOP amplitude mapping. c, OOP phase mapping.

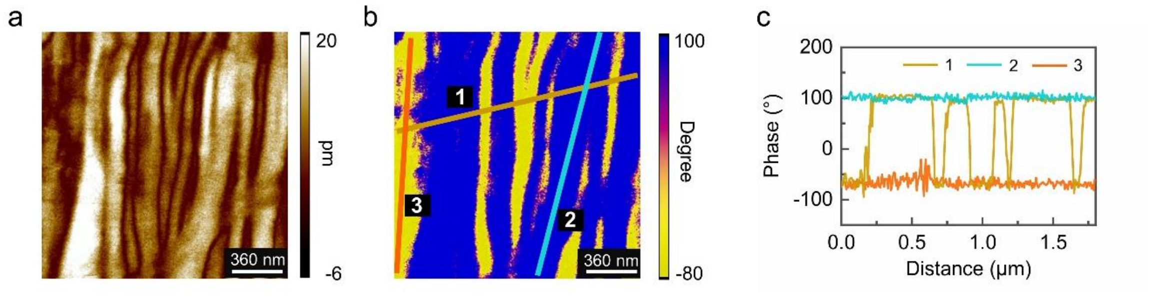

Supplementary Figure 16. PFM measurements of collagen films. a, PFM IP amplitude mapping, b, IP phase mapping. c, Cross-sectional profiles of piezoelectric polarization responses along the line 1,2 and 3 in phase mapping in b.

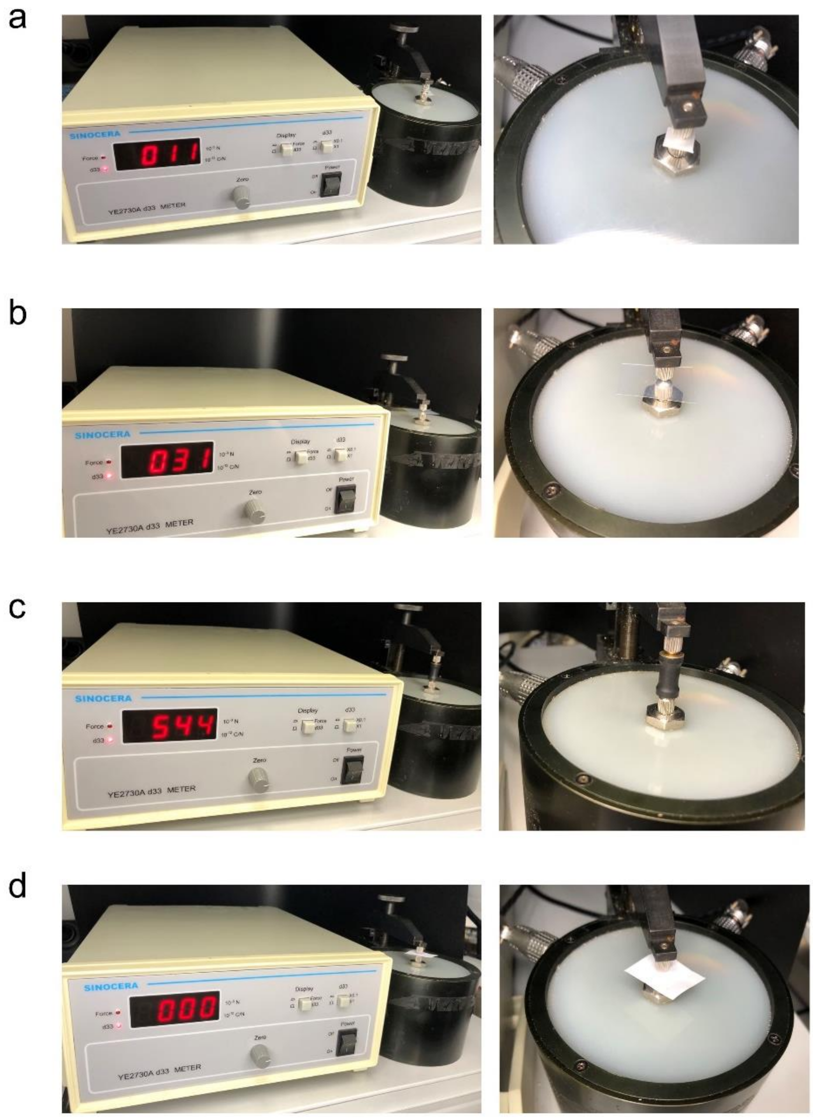

Supplementary Figure 17. Macroscopic piezoelectricity of the β-glycine nanocrystalline films on aluminum foil (a), commercial PVDF film (b), PZT for calibration (c), and non-piezoelectric printer paper (d) measured by a commercial d_{33} meter.

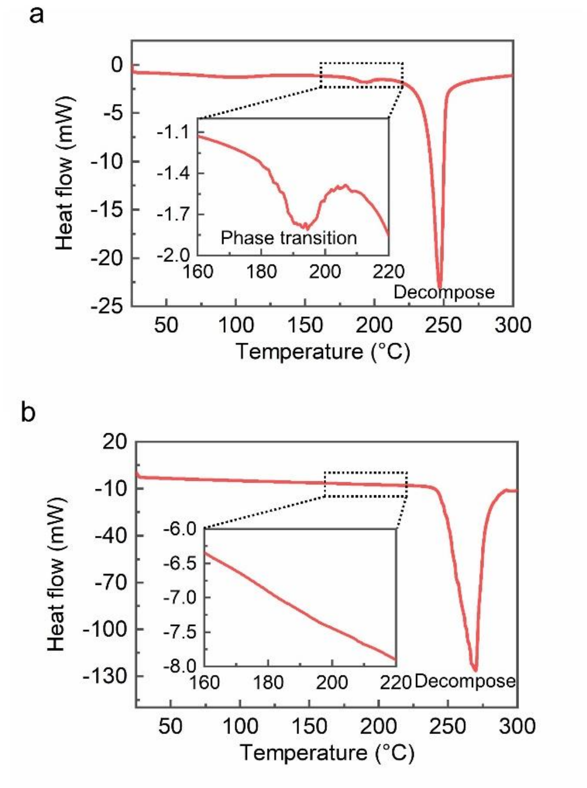

Supplementary Figure 18. DSC measurements of γ-glycine crystals (a) and α-glycine crystals (b). The inset figures are the enlarged drawing of DSC curve between the temperature of 160 °C and 220 °C.

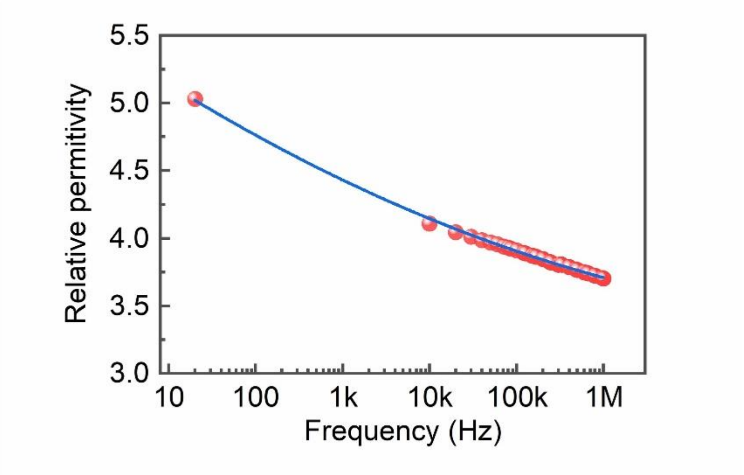

Supplementary Figure 19. Relative permittivity of β-glycine nanocrystalline films with a thickness of 1.3 μm.

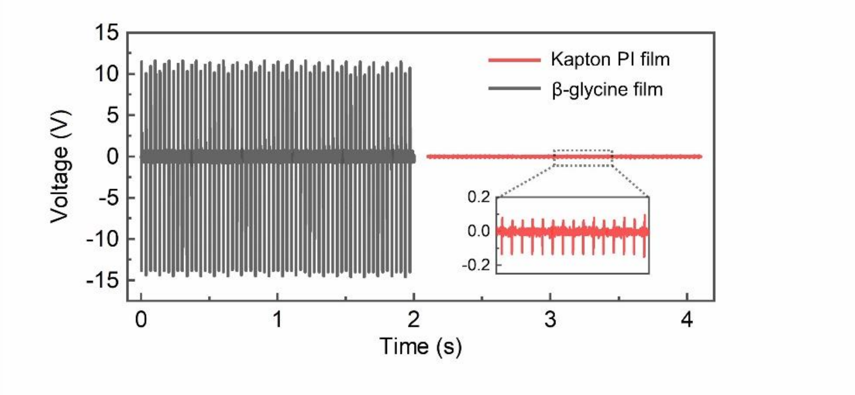

Supplementary Figure 20. Comparison of the open-circuit voltage measurements of the piezoelectric device of the β-glycine nanocrystalline films and the control device of the non-piezoelectric Kapton PI film.

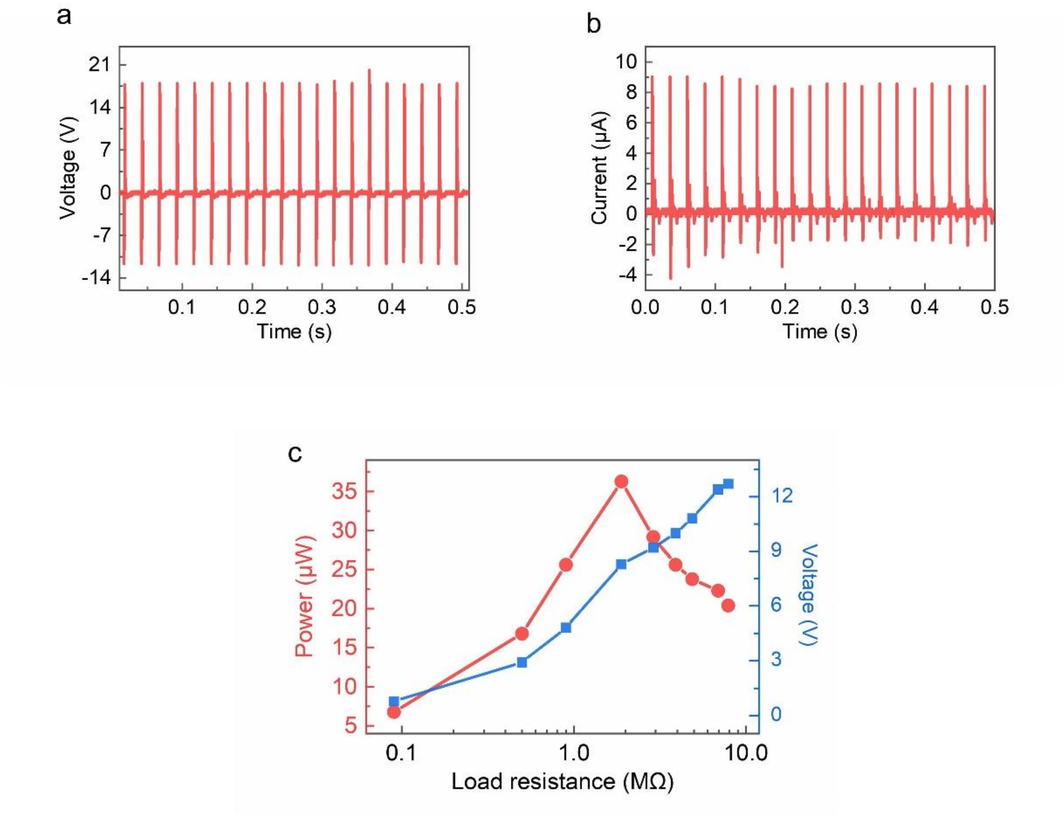

Supplementary Figure 21. PVDF film-based piezoelectric device measurements. a, The measured open-circuit voltage. b, The measured short-circuit current. c, Dependence of the power output of the piezoelectric device on the resistance of the external load.

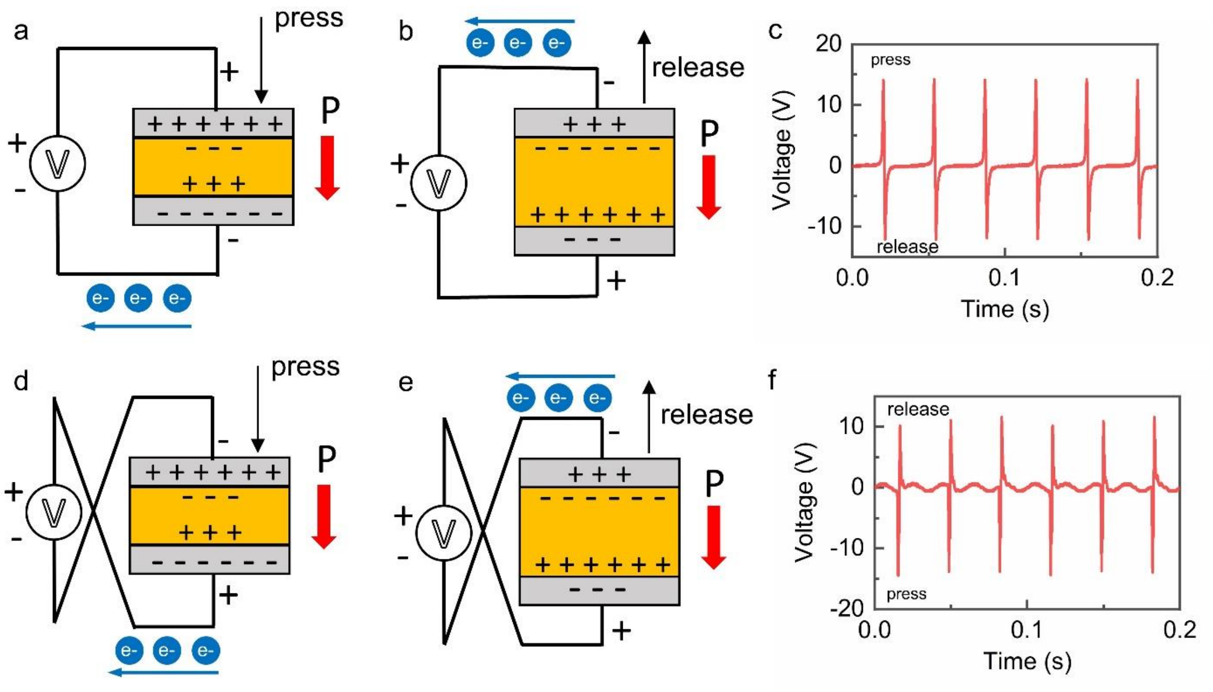

Supplementary Figure 22. Open-circuit voltage measurements of the piezoelectric device of β-glycine nanocrystalline films. Schematics of the measurements with the forward connection in press state (a) and release state (b). c, The measured open-circuit voltage in the forward connection. Schematics of the measurements with the reverse connection in press state (d) and release state (e). f, The measured open-circuit voltage in the reverse connection. The blue arrow denotes the electron flow direction. The red arrow P represents the polarization direction of β-glycine nanocrystalline films.

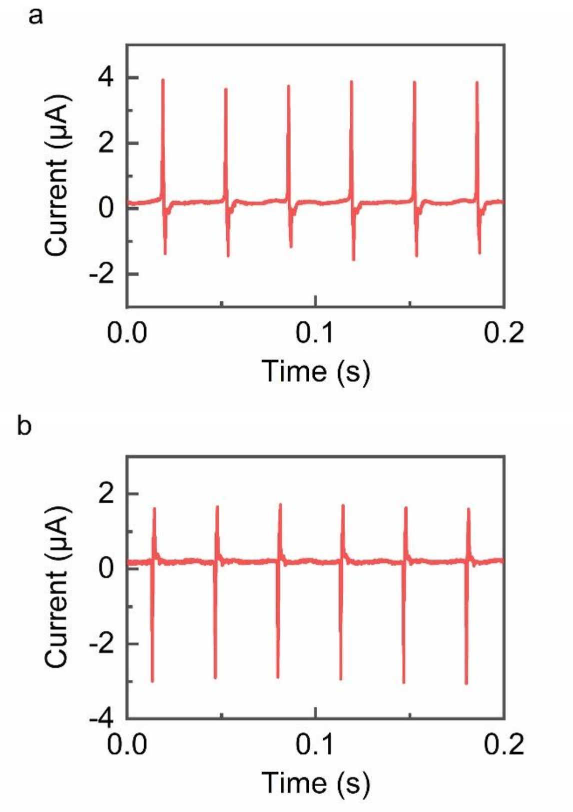

Supplementary Figure 23. Short-circuit current measurements of the piezoelectric device of β-glycine nanocrystalline films in the forward connection (a) and the reverse connection (b).

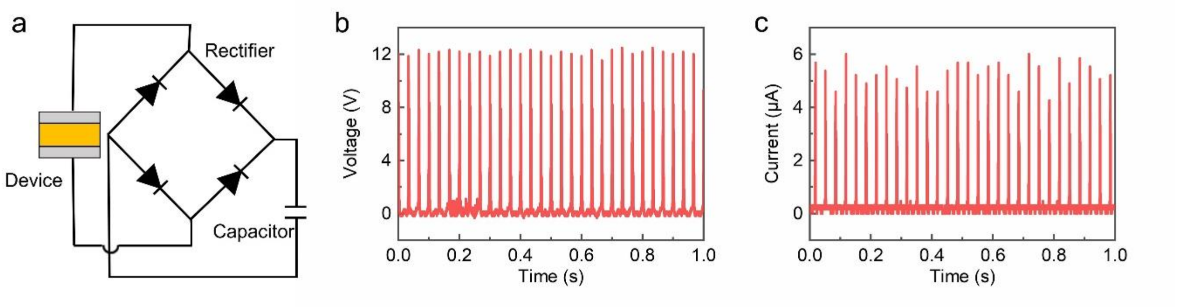

Supplementary Figure 24. a, Circuit diagram of storing the electricity produced from the piezoelectric device of β-glycine nanocrystalline films. b, Rectified voltage signals under the compression force at a frequency of 30 Hz. c, Corresponding rectified current signals.

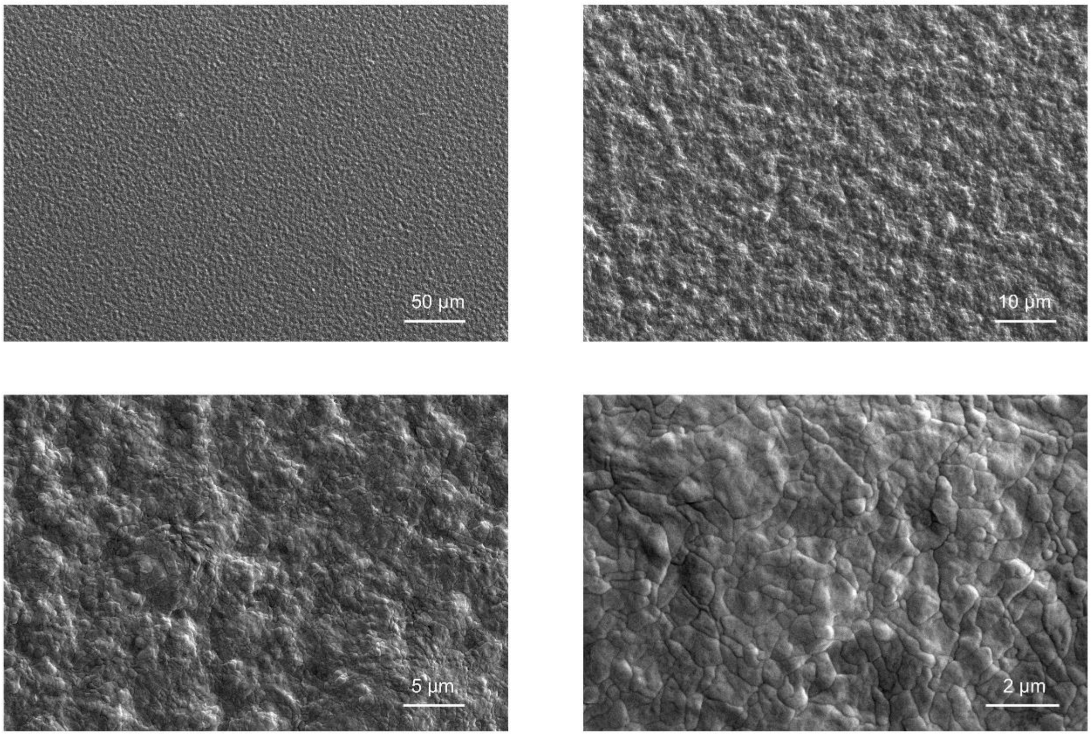

Supplementary Figure 25. Surface topography SEM image of the films after durability tests of 24,000 compressing cycles.

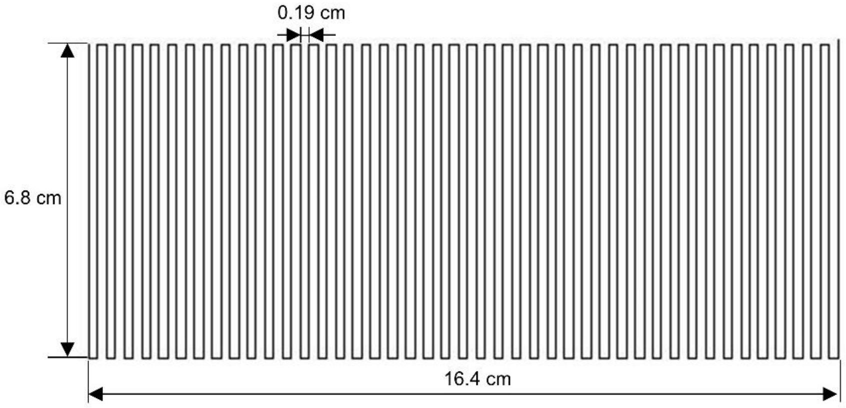

Supplementary Figure 26. The X-Y translational stage movement route with a moving speed of 82 cm min^{-1} for determining the β-glycine film depositing speed.

Supplementary Tables

Supplementary Table 1: List of piezoelectric coefficients and measurement method of representative piezoelectric biomaterials, which are plotted in Fig. 4d.

Material	Piezoelectric coefficient (pm V^{-1})	Measurement method	Reference
Wood	0.1	Stress-induced electric polarization measurements (direct piezoelectric effect)	9
Bone	0.2	Stress-induced electric polarization measurements (direct piezoelectric effect)	10
Cellulose	1.3	Electric field-induced strain measurements (inverse piezoelectric effect)	11
Silk	1.5	Stress-induced current measurements (direct piezoelectric effect)	12
Collagen	2.6	PFM (inverse piezoelectric effect)	13
ZnO	3.0	PFM (inverse piezoelectric effect)	14
Virus	3.9	PFM (inverse piezoelectric effect)	15
Chitin	4.0	PFM (inverse piezoelectric effect)	16
γ-glycine-PVA film	5.3	Quasi-static d_{33} meter (direct piezoelectric effect)	17
**β-glycine nanocrystalline film (this work)**	**11.2**	PFM (inverse piezoelectric effect)	

Supplementary Table 2: List of piezoelectric voltage coefficients and curie temperature of representative piezoelectric materials, which are plotted in Fig. 5g.

Types	Material	Curie temperature (°C)	g33 (10^{-3} V m/N)	Reference
Biomaterial	cellulose	132	27	11,18,19
	Chitin	75	108	16,20,21
	collagen	60	47	13,22,23
	virus	95	73	15,24
	silk	178	56	12,18,25
Synthetic polymer	PVDF	130	286	26–28
	PLLA	60	108	29–31
PZT	PZT-5H	193	20	32
	Commercial PZT (Navi V 854)	250	26	33
	Commercial PZT (Navi VI 855)	200	21	33
BTO	BTO	120	11	34
	Ba(Ti0.8Zr0.2)O3－(Ba0.7Ca0.3)TiO3	93	22	35
	0.82Ba(Ti0.89Sn0.11)O3–x0.18(Ba0.7Ca0.3)TiO3	60	26	36
	0.84BaTiO3–0.16(0.4CaTiO3–0.6BaSnO3)	63	21	37
Inorganic single crystal	PMNT29	137	31	38
	PMN-PZT	216	36	38
	Sm-PMN-PT	115	35	39
	BTO	120	58	40
Glycine	bulk β-glycine crystal	67	296	41
	γ-glycine-PVA film	195	158	17
	**β-glycine nanocrystalline film (This work)**	192	252	

Supplementary Table 3: Comparison of the output performances of this work with those of existing reports. (* denotes the power density is calculated by multiplying the open-circuit voltage and short-circuit current density.)

**Type**	**Voltage (V)**	**Current (μA)**	**Power density (μW cm^{-2})**	**Reference**
Virus	0.4	0.004	0.016*	15
	0.3	0.015	0.00029	42
	2.8	0.12	0.24	43
	1	0.09	0.08	44
Diphenylalanine	1.4	0.04	0.003	45
	2.8	0.0374	0.008	46
	0.6	0.007	0.000007	47
	1.8	0.07	0.008	48
Wood	0.9	0.017	0.0052*	49
	0.7	0.007	0.0006	50
Chitin	1	0.3	0.184	16
Peptide	1.2	0.05	0.122*	51
	1.2	0.0018	0.0015*	52
Amino acid	0.45	-	-	41
	0.8	-	-	53
	0.18	-	-	54
	2.5	0.2	0.83*	17
**This work**	**14.5**	**4**	**3.61**	

Supplementary Table 4: Comparison of the power densities of this work with other renewable energy harvesting methods.

Type	Power density (μW cm^{-2})	Reference
Solar	663	[55]
Geothermal	224	[55]
Wind	284	[55]
Hydro	14	[55]
Thermoelectric	40	[56]
Biomass	8	[55]
Electromagnetic	4	[56]
**This work (mechanical energy harvesting)**	**3.61**	

Supplementary References

1. Jaworek, A. & Krupa, A. *CLASSIFICATION OF THE MODES OF EHD SPRAYING*. *J. Aerosol Sci* vol. 30 (1999).
2. Chen, D. R. & Pui, D. Y. H. Experimental investigation of scaling laws for electrospraying: Dielectric constant effect. *Aerosol Science and Technology* **27**, 367–380 (1997).
3. Ward, M. D. Perils of Polymorphism: Size Matters. *Israel Journal of Chemistry* vol. 57 82–92 Preprint at https://doi.org/10.1002/ijch.201600071 (2017).
4. Mullin, J. *Crystallization*. (2001).
5. Bernstein, J. *Polymorphism in Molecular Crystals 2e*. (2020).
6. Rault, J., Neffati, R. & Judeinstein, P. Melting of ice in porous glass: Why water and solvents confined in small pores do not crystallize? *European Physical Journal B* **36**, 627–637 (2003).
7. Unruh, K. M., Huber, T. E. & Huber, C. A. *Melting and freezing behavior of indium metal in porous glasses*. *PHYSICAL REVIEW B* vol. 48 (1993).
8. Jackson, C. L. & McKenna, G. B. The melting behavior of organic materials confined in porous solids. *J Chem Phys* **93**, 9002–9011 (1990).
9. Fukada, E. Piezoelectricity of Wood. *J Physical Soc Japan* **10**, 149–154 (1955).
10. Fukada, E. & Yasuda, I. On the piezoelectric effect of bone. *J Physical Soc Japan* **12**, 1158–1162 (1957).
11. García, Y., Ruiz-Blanco, Y. B., Marrero-Ponce, Y. & Sotomayor-Torres, C. M. Orthotropic Piezoelectricity in 2D Nanocellulose. *Sci Rep* **6**, (2016).
12. Yucel, T., Cebe, P. & Kaplan, D. L. Structural origins of silk piezoelectricity. *Adv Funct Mater* **21**, 779–785 (2011).
13. Denning, D. *et al.* Piezoelectric Tensor of Collagen Fibrils Determined at the Nanoscale. *ACS Biomater Sci Eng* **3**, 929–935 (2017).
14. Scrymgeour, D. A., Sounart, T. L., Simmons, N. C. & Hsu, J. W. P. Polarity and piezoelectric response of solution grown zinc oxide nanocrystals on silver. *J Appl Phys* **101**, (2007).
15. Lee, B. Y. *et al.* Virus-based piezoelectric energy generation. *Nat Nanotechnol* **7**, 351–356 (2012).
16. Kim, K. *et al.* Biodegradable, electro-active chitin nanofiber films for flexible piezoelectric transducers. *Nano Energy* **48**, 275–283 (2018).
17. Yang, F. *et al. Wafer-scale heterostructured piezoelectric bio-organic thin films*. http://science.sciencemag.org/.
18. Clipper Controls Inc. Dielectric Constant Values. *https://www.clippercontrols.com/pages/Dielectric-Constant-Values.html* (2022).
19. Picker, K. M. & Hoag, S. W. Characterization of the thermal properties of microcrystalline cellulose by modulated temperature differential scanning calorimetry. *J Pharm Sci* **91**, 342–349 (2002).
20. Mitra, T. *et al.* Preparation and characterization of a thermostable and biodegradable biopolymers using natural cross-linker. *Int J Biol Macromol* **48**, 276–285 (2011).
21. Saravanan, D., Gomathi, T. & Sudha, P. N. *Comparative Study of Thermal Stability Using Natural Polymer Blend by Cross Linking*. *Archives of Applied Science Research* vol. 3 www.scholarsresearchlibrary.com (2011).
22. Marzec, E. & Pietrucha, K. The effect of different methods of cross-linking of collagen on its

dielectric properties. *Biophys Chem* **132**, 89–96 (2008).
23. Muszyński, S. *et al.* Effects of replacing soybean meal with chickpea seeds in the diet on mechanical and thermal properties of tendon tissue in broiler chicken. *Poult Sci* **97**, 695–700 (2018).
24. Branston, S. D., Stanley, E. C., Ward, J. M. & Keshavarz-Moore, E. Determination of the survival of bacteriophage M13 from chemical and physical challenges to assist in its sustainable bioprocessing. *Biotechnology and Bioprocess Engineering* **18**, 560–566 (2013).
25. Hu, X., Lu, Q., Kaplan, D. L. & Cebe, P. Microphase separation controlled β-sheet crystallization kinetics in fibrous proteins. *Macromolecules* **42**, 2079–2087 (2009).
26. Li, M. *et al.* Revisiting the δ-phase of poly(vinylidene fluoride) for solution-processed ferroelectric thin films. *Nat Mater* **12**, 433–438 (2013).
27. Lovinger, A. J., Davis, D. D., Cais, R. E. & Kometani, J. M. *The role of molecular defects on the structure and phase transitions of poly(vinylidene fluoride)*. (1987).
28. Chen, X. G. *et al.* Two-Dimensional Layered Perovskite Ferroelectric with Giant Piezoelectric Voltage Coefficient. *J Am Chem Soc* **142**, 1077–1082 (2020).
29. Sarasua, J. R. *et al.* Crystallinity assessment and in vitro cytotoxicity of polylactide scaffolds for biomedical applications. *J Mater Sci Mater Med* **22**, 2513–2523 (2011).
30. Zhao, G. *et al.* Electrospun Poly(l-Lactic Acid) Nanofibers for Nanogenerator and Diagnostic Sensor Applications. *Macromol Mater Eng* **302**, 1–7 (2017).
31. Hikosaka, S., Ishikawa, H. & Ohki, Y. Effects of crystallinity on dielectric properties of poly(L-lactide). *Electronics and Communications in Japan* **94**, 1–8 (2011).
32. Yang, Z., Zhou, S., Zu, J. & Inman, D. High-Performance Piezoelectric Energy Harvesters and Their Applications. *Joule* **2**, 642–697 (2018).
33. APC International Ltd. PHYSICAL AND PIEZOELECTRIC PROPERTIES OF APC MATERIALS. *https://www.americanpiezo.com/apc-materials/piezoelectric-properties.html* (2021).
34. Bechmann, R. Elastic, Piezoelectric, and Dielectric Constants of Polarized Barium Titanate Ceramics and Some Applications of the Piezoelectric Equations. *Journal of the Acoustical Society of America* **28**, 347–350 (1956).
35. Liu, W. & Ren, X. Large piezoelectric effect in Pb-free ceramics. *Phys Rev Lett* **103**, (2009).
36. Zhao, C. *et al.* Practical High Piezoelectricity in Barium Titanate Ceramics Utilizing Multiphase Convergence with Broad Structural Flexibility. *J Am Chem Soc* **140**, 15252–15260 (2018).
37. Zhu, L. F., Zhang, B. P., Zhao, L. & Li, J. F. High piezoelectricity of BaTiO3-CaTiO3-BaSnO 3 lead-free ceramics. *J Mater Chem C Mater* **2**, 4764–4771 (2014).
38. Zhang, S., Lee, S. M., Kim, D. H., Lee, H. Y. & Shrout, T. R. Temperature dependence of the dielectric, piezoelectric, and elastic constants for Pb (Mg13 Nb23) O3 -PbZr O3 -PbTi O3 piezocrystals. *J Appl Phys* **102**, (2007).
39. Li, F. *et al. Giant piezoelectricity of Sm-doped Pb(Mg 1/3 Nb 2/3)O 3-PbTiO 3 single crystals*. https://www.science.org.
40. Newnham, R. E., Bowen, L. J., Klicker, K. A. & Cross, L. E. *Composite Piezoelectric Transducers*.
41. Guerin, S. *et al.* Control of piezoelectricity in amino acids by supramolecular packing. *Nat Mater* **17**, 180–186 (2018).
42. Shin, D. M. *et al.* Bioinspired piezoelectric nanogenerators based on vertically aligned phage nanopillars. *Energy Environ Sci* **8**, 3198–3203 (2015).
43. Lee, J. H. *et al.* Vertical Self-Assembly of Polarized Phage Nanostructure for Energy Harvesting. *Nano Lett* **19**, 2661–2667 (2019).

44. Heo, K. *et al.* Transient self-templating assembly of M13 bacteriophage for enhanced biopiezoelectric devices. *Nano Energy* **56**, 716–723 (2019).
45. Nguyen, V., Zhu, R., Jenkins, K. & Yang, R. Self-assembly of diphenylalanine peptide with controlled polarization for power generation. *Nat Commun* **7**, 1–6 (2016).
46. Lee, J. H. *et al.* Diphenylalanine peptide nanotube energy harvesters. *ACS Nano* **12**, 8138–8144 (2018).
47. Jenkins, K., Kelly, S., Nguyen, V., Wu, Y. & Yang, R. Piezoelectric diphenylalanine peptide for greatly improved flexible nanogenerators. *Nano Energy* **51**, 317–323 (2018).
48. Tao, Z. *et al.* Diphenylalanine-based degradable piezoelectric nanogenerators enabled by polylactic acid polymer-assisted transfer. *Nano Energy* **88**, 106229 (2021).
49. Sun, J. *et al.* Enhanced mechanical energy conversion with selectively decayed wood. *Sci Adv* **7**, 1–8 (2021).
50. Sun, J. *et al.* Sustainable and biodegradable wood sponge piezoelectric nanogenerator for sensing and energy harvesting applications. *ACS Nano* **14**, 14665–14674 (2020).
51. Bera, S. *et al.* Molecular engineering of piezoelectricity in collagen-mimicking peptide assemblies. *Nat Commun* **12**, (2021).
52. Tao, K. *et al.* Bioinspired Stable and Photoluminescent Assemblies for Power Generation. *Advanced Materials* **31**, 1–7 (2019).
53. Guerin, S. *et al.* Racemic Amino Acid Piezoelectric Transducer. *Phys Rev Lett* **122**, 47701 (2019).
54. Hosseini, E. S., Manjakkal, L., Shakthivel, D. & Dahiya, R. Glycine-Chitosan-Based Flexible Biodegradable Piezoelectric Pressure Sensor. *ACS Appl Mater Interfaces* **12**, 9008–9016 (2020).
55. van Zalk, J. & Behrens, P. The spatial extent of renewable and non-renewable power generation: A review and meta-analysis of power densities and their application in the U.S. *Energy Policy* **123**, 83–91 (2018).
56. Dziadak, B., Makowski, Ł. & Michalski, A. Survey of energy harvesting systems for wireless sensor networks in environmental monitoring. *Metrology and Measurement Systems* **23**, 495–512 (2016).
