# Supplementary material for: Active self-assembly of piezoelectric biomolecular films via synergistic nanoconfinement and in-situ poling
Source: Nat Commun. 2023 Jul 11;14:4094. doi: 10.1038/s41467-023-39692-y (PMC10336032; doi:10.1038/s41467-023-39692-y)
Supplement: Supplementary file 3 — Description of Additional Supplementary File [file 41467_2023_39692_MOESM3_ESM.pdf]

Description of Additional Supplementary File

Movie Legends

Supplementary Movie 1: Electrohydrodynamic spay process for the β-glycine nanocrystalline film synthesis.

Supplementary Movie 2: The β-glycine nanocrystalline film-based piezoelectric device produces electricity under a tapping force, and the power is high enough to light up three LEDs.
